# Major Depression as a Catalyst for Nonalcoholic Fatty Liver Disease: Insights From a Comprehensive Mendelian Randomization Study

**DOI:** 10.1155/cjgh/6632867

**Published:** 2025-08-30

**Authors:** Yinshi Su, Shuangzhe Lin, Ling She, Jingping Xiong, Yongnian Ding, Yan Zhang, Yuanwen Chen

**Affiliations:** ^1^ Department of Gastroenterology, Huadong Hospital, Fudan University, Shanghai, 200040, China, fudan.edu.cn; ^2^ Department of Gastroenterology, The Second Affiliated Hospital of Xinjiang Medical University, Ürümqi, Xinjiang, 830028, China, xjmu.edu.cn; ^3^ Department of Gastroenterology, The Seventh Affiliated Hospital of Xinjiang Medical University, Ürümqi, Xinjiang, 830028, China; ^4^ Department of Gerontology, Huadong Hospital, Fudan University, Shanghai, China, fudan.edu.cn

**Keywords:** body mass index, major depression, mediation, Mendelian randomization, mental disorders, metabolic dysfunction-associated steatotic liver disease (MASLD), nonalcoholic fatty liver disease (NAFLD), obesity, waist-to-hip ratio

## Abstract

**Background:** This study aimed to investigate the causal association between mental disorders and nonalcoholic fatty liver disease (NAFLD; now termed metabolic dysfunction‐associated steatotic liver disease, MASLD) using Mendelian randomization (MR).

**Methods:** A bidirectional two‐sample MR approach was employed to evaluate the causal relationship between NAFLD and eight mental disorders: major depression, anxiety, bipolar disorder, schizophrenia, autism, post‐traumatic stress disorder (PTSD), attention deficit hyperactivity disorder (ADHD), and eating disorders. The inverse‐variance weighted (IVW) method was utilized to estimate causal effects, supported by MR‐CAUSE and other sensitivity analyses to address pleiotropy and heterogeneity. Instrumental variables from genome‐wide association studies were applied, and initial associations were examined using linkage disequilibrium score (LDSC) regression analysis. Furthermore, mediation analysis was conducted to identify potential mediators.

**Results:** The MR analysis revealed significant positive associations between NAFLD occurrence and major depression (odds ratio [OR] = 1.294, *p* = 0.003), bipolar disorder (OR = 0.895, *p* = 0.004), and autism (OR = 1.118, *p* = 0.005). However, only the putative causal association of major depression on NAFLD remained statistically significant in further validation, including MR‐CAUSE and sensitivity analyses, and was not attributable to linkage disequilibrium. No causal effect of NAFLD on mental disorders was found in reverse MR analysis. In LDSC regression, significant positive associations with NAFLD occurrence were observed for major depression (Rg = 0.538, *p* = 1.36*E* − 07), ADHD (Rg = 0.798, *p* = 1.10*E* − 08), and PTSD (Rg = 0.579, *p* = 0.009). Mediation analysis identified body mass index (39.33%, *p* = 0.017), waist‐to‐hip ratio (8.09%, *p* = 0.026), triglycerides (39.33%, *p* = 0.017), and serum concentration of large very low‐density lipoprotein (VLDL) particles (8.76%, *p* = 0.032) as mediators of the causal effect of major depression on NAFLD occurrence.

**Conclusion:** This study suggests a potential causal link between major depression and the development of NAFLD, underscoring the importance of considering major depression in the management of NAFLD patients.

## 1. Introduction

Nonalcoholic fatty liver disease (NAFLD), a condition recently redefined as metabolic dysfunction‐associated steatotic liver disease (MASLD) to better reflect its pathogenesis [1], is a liver disease characterized by varying degrees of hepatic steatosis and inflammation and has become a leading cause of chronic liver disease worldwide [2]. Throughout this manuscript, the new term MASLD will be used, while acknowledging that the legacy Genome‐Wide Association Study (GWAS) datasets for our analysis were defined under the previous NAFLD criteria. Approximately 30% of the global population is affected by MASLD [3]. More importantly, NAFLD patients can develop liver fibrosis, cirrhosis, or even liver carcinoma during disease progression [4]. It can also increase the risk of cardiovascular disease, type 2 diabetes mellitus (T2DM), osteoporosis, and chronic kidney disease [5]. Therefore, the continuous increase in NAFLD incidence has greatly burdened economic and social development worldwide [6]. Although NAFLD has been traditionally regarded as the hepatic manifestation of metabolic syndrome (MetS) [7], the pathophysiology of NAFLD, including disease causes, risk factors, and pathological mechanisms, has not been fully elucidated, contributing to difficulties in effective treatment and prevention of NAFLD.

Mental disorders are also highly prevalent diseases and are amongst the top global disease burdens [8]. It is estimated that approximately 1 in 8 people, or 970 million people worldwide, suffer from a mental disorder [9]. Mental disorders per se are harmful, leading to shorter lifespans and higher all‐cause mortality rates [10]. But more importantly, it has been proposed that mental disorders can have reciprocal interaction with physical disorders: Physical disorders may contribute to mental disorders, while mental disorders may also increase the risk of physical disorders [11, 12]. This may stem from the close interaction between the nervous system and metabolic, immunological, or endocrinological factors [13]. Interestingly, the comorbidity of NAFLD and mental disorders has been frequently found, and a significantly higher risk of NAFLD occurrence in patients with mental illnesses has also been reported in previous observational studies [14–16].

However, the mechanisms between NAFLD and mental disorders still need to be elucidated. Chronic psychological stress and mental disorders, such as depression and anxiety, can activate the body’s stress response system, including the hypothalamic‐pituitary‐adrenal (HPA) axis, which is related to lipid metabolism [17, 18]. In addition, mental disorders are associated with insulin resistance, a key pathological feature of NAFLD; long‐term psychological stress can affect insulin sensitivity, leading to abnormal blood sugar control and promoting liver fat deposition [19]. Furthermore, mental disorders are related to changes in the gut microbiome, a microbial ecosystem closely related to human health [20]. Abnormalities in the gut microbiota of patients with mental disorders may affect energy metabolism, increase the production of endotoxins, enhance intestinal permeability, cause endotoxemia, and exacerbate liver inflammation and fat accumulation [21]. Therefore, mental disorders may affect NAFLD by influencing the HPA axis, insulin resistance, or gut microbiota. Considering the complicated involvement of metabolic and immunologic disorders in NAFLD pathogenesis [22], the close epidemiological association between NAFLD and mental disorders might stem from causal effects from mental disorders on NAFLD incidence. Therefore, there is a need to further clarify the interaction between NAFLD and mental disorders.

Mendelian randomization (MR) is a novel statistical method for exploration of disease causality. By using genetic variants as a tool for exposure, MR is not subject to the influence of confounding factors or reverse causation. Genetic association, as genes are randomly allocated at conception [23]. In this study, we systematically performed a two‐sample MR analysis to investigate the causal association of mental disorders with NAFLD. Linkage disequilibrium score (LDSC) regression analysis was used to explore their association preliminarily. For further exploration of the underlying mechanism, a two‐step MR was also conducted to investigate the mediating factors because these factors may be crucial in the prevention and treatment of NAFLD.

## 2. Materials and Methods

### 2.1. Study Design

The present study was reported under the guidance of Strengthening the Reporting of Observational Studies in Epidemiology Using Mendelian Randomization: The STROBE‐MR Statement [24]. In the preliminary analysis, the two‐sample MR analysis was performed to explore the potential causal effects of mental disorders on NAFLD. The mental disorders we studied include major depression, anxiety, bipolar disorder, schizophrenia, autism, post‐traumatic stress disorder (PTSD), attention deficit hyperactivity disorder (ADHD), and eating disorders. Outcome data for discovery analysis were obtained from the large NAFLD GWAS meta‐analysis conducted by Ghodsian et al. [25]. Furthermore, for the replication analysis, data were retrieved from the FinnGen Consortium (R8 release) [26].

In addition, it has been proposed that NAFLD may also contribute to mental disorders [15]. To explore the causal relationship between mental disorders and the occurrence of NAFLD, a reverse MR analysis was conducted. To ensure the reliability of MR study results, three major assumptions of MR research [27] were considered when we designed this study.

**Assumption 1.**

The selected IVs are robustly associated with the exposure.

**Assumption 2.**

Exposure‐related IVs are not influenced by confounding factors.

**Assumption 3.**

The selected IVs only affect the specific outcome through exposure.

**Figure 1:**
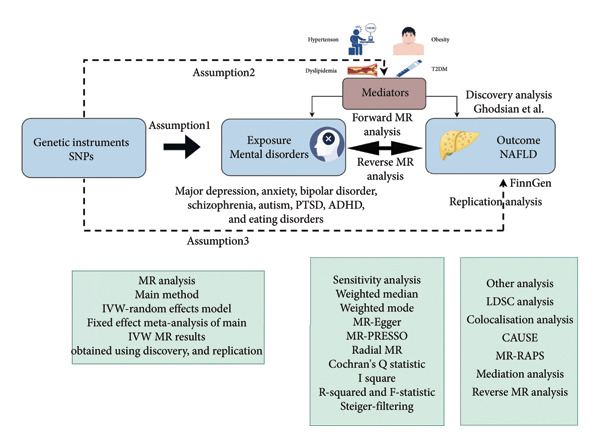
Overview of the MR study design. Abbreviations: SNP, single‐nucleotide polymorphism; MR, Mendelian randomization; PTSD, post‐traumatic stress disorder; ADHD, attention deficit hyperactivity disorder; T2DM, Type 2 diabetes mellitus; NAFLD, nonalcoholic fatty liver disease; MR‐PRESSO, Mendelian randomization–pleiotropy residual sum and outlier; LDSC, linkage disequilibrium score regression; CAUSE, causal analysis using summary effect estimates; MR‐RAPS, MR–robust adjusted profile score.

The overall design of this study is illustrated in Figure 1.

### 2.2. Data Source

Our two‐sample MR study was based on publicly available data from summary‐level GWAS consortiums. The detailed information on the GWAS data used in this study is presented in Table 1. While the data pertaining to mental disorders were extracted from prior scholarly works, the NAFLD data were predominantly obtained from the FinnGen consortium. The mediators’ data were sourced from the IEU OpenGWAS repository. The dataset for our investigation was derived from publicly accessible registry data, sourced from studies that had rigorously adhered to the ethical principles established in the Declaration of Helsinki and conformed to Good Clinical Practice guidelines. Given this adherence to stringent ethical and clinical standards in the original studies, no further ethical approval was required for the current research.

**Table 1:** Characteristics of all GWAS databases used in this study.

Traits	Sample size	Population	Consortium	PMID or URL
*Mental disorders*				
Major depression	500,199(170,756cases/329,443 controls)	Europeans	PGC	3,07,18,901
Attention deficit hyperactivity disorder (ADHD)	55,374(20,183cases/35,191 controls)	Europeans	PGC	30,478,444
Anxiety disorder	17,310(5580cases/11730 controls)	Europeans	PGC	26,754,954
Post‐traumatic stress disorder (PTSD)	174,659(23,212cases/151447 controls)	Europeans	PGC	31,594,949
Bipolar disorder	413,466(41,917cases/371549 controls)	Europeans	PGC	34,002,096
Autism spectrum disorder	46,351(18,382cases/27969 controls)	Europeans	PGC	28,540,026
Schizophrenia	130,644(53,386cases/77258 controls)	Europeans	PGC	35,396,580
Eating disorders	72,517(16,992cases/55525 controls)	Europeans	PGC	31,308,545

*NAFLD*				
NAFLD (studies for discovery analysis)	778,614(8434cases/770180 controls)	Europeans	eMERGE, UK Biobank, FinnGen, and Estonian Biobank	34,841,290
NAFLD (studies for replication analysis)	342,499(1908cases/340591 controls)	Europeans	FinnGen consortium	https://r8.finngen.fi/

*Mediator: Dyslipidemia traits*				
Triglycerides	343,992	European	IEU OpenGWAS	34594039/GWAS ID: ebi‐a‐GCST90018975
Total cholesterol levels	344,278	European	IEU OpenGWAS	34594039/GWAS ID: ebi‐a‐GCST90018974
HDL cholesterol	400,754	European	IEU OpenGWAS	34226706/GWAS ID:ebi‐a‐GCST90025956
LDL cholesterol	343,621	European	IEU OpenGWAS	34594039/GWAS ID:ebi‐a‐GCST90018961
VLDL cholesterol	115,082	European	IEU OpenGWAS	34594039/GWAS ID:ebi‐a‐GCST90092996
Concentration of small VLDL particles	115,078	European	IEU OpenGWAS	GWAS ID:met‐d‐S_VLDL_P
Concentration of small LDL particles	115,078	European	IEU OpenGWAS	GWAS ID:met‐d‐S_LDL_P
Concentration of small HDL particles	115,078	European	IEU OpenGWAS	GWAS ID:met‐d‐S_HDL_P
Concentration of medium VLDL particles	115,078	European	IEU OpenGWAS	GWAS ID:met‐d‐M_VLDL_P
Concentration of medium LDL particles	115,078	European	IEU OpenGWAS	GWAS ID:met‐d‐M_LDL_P
Concentration of medium HDL particles	115,078	European	IEU OpenGWAS	GWAS ID:met‐d‐M_HDL_P
Concentration of large VLDL particles	115,078	European	IEU OpenGWAS	GWAS ID:met‐d‐L_VLDL_P
Concentration of large VLDL particles	115,078	European	IEU OpenGWAS	GWAS ID:met‐d‐L_LDL_P
Concentration of large VLDL particles	115,078	European	IEU OpenGWAS	GWAS ID:met‐d‐L_HDL_P

*Mediator: Obesity*				
Body mass index (BMI)	457,756	European	IEU OpenGWAS	34226706/GWAS ID:ebi‐a‐GCST90025994
Waist circumference	462,166	European	IEU OpenGWAS	GWAS ID:ukb‐b‐9405
Body fat percentage	454,633	European	IEU OpenGWAS	29892013/GWAS ID:ukb‐b‐8909
Waist‐hip ratio	502,773	European	IEU OpenGWAS	29892013/GWAS ID:ebi‐a‐GCST90029009

*Mediator:Hypertenson*				
Systolic blood pressure	340,159	European	IEU OpenGWAS	34594039/GWAS ID:ebi‐a‐GCST90018972
Diastolic blood pressure	757,601	European	IEU OpenGWAS	30224653/GWAS ID:ieu‐b‐39

*Mediator: T2DM*				
Fasting glucose	200,622	European	IEU OpenGWAS	34059833/GWAS ID:ebi‐a‐GCST90002232
Fasting insulin	151,013	European	IEU OpenGWAS	34059833/GWAS ID:ebi‐a‐GCST90002238
Glycated hemoglobin HbA1c	389,889	European	IEU OpenGWAS	34017140/GWAS ID:ebi‐a‐GCST90014006

### 2.3. Instrumental Variable Selection

The genome‐wide significance threshold for selecting valid IVs was set at *p* < 5 × 10^−8^ for major depression, ADHD, bipolar disorder, schizophrenia, and eating disorders, as these conditions had a sufficient number of SNPs meeting this stringent criterion. However, due to an insufficient number of available SNPs for anxiety, PTSD, and autism at the genome‐wide significance level (*p* < 5 × 10^−8^), we applied a more lenient, yet widely accepted, threshold of *p* < 5 × 10^−6^ to ensure a sufficient number of IVs for these conditions, as previously described [28]. To mitigate the risk of weak instrument bias potentially introduced by this relaxed threshold, the strength of each selected instrument was assessed using F‐statistics. The mean F‐statistics for the instrument sets were 24.6 for anxiety, 22.8 for PTSD, and 24.3 for autism. As all individual F‐statistics substantially exceeded the conventional threshold of 10, the selected instruments were considered sufficiently strong, minimizing the risk of weak instrument bias (details in Supporting Table 1). All selected SNPs were further clumped to a linkage disequilibrium threshold of *r* ^2^ < 0.001 within a 10,000 kb window to select independent genetic variants. Similarly, in the reverse MR analysis, a threshold of *p* < 5*e* ^−6^ was set for the NAFLD to obtain sufficient SNPs. Furthermore, we removed SNPs that were palindromic or associated with incompatible alleles. When the SNPs were not available for outcome, higher linkage disequilibrium (*R* ^2^ ≥ 0.8) was employed to substitute for them through the CEU reference population from the 1000 Genomes Project.

For the MR analysis, SNPs served as instrumental variables to investigate the potential causal relationship between major depression and NAFLD. The list of instrumental variables used for major depression is provided in Supporting Table 2.

To validate the findings from the initial analysis, we conducted an independent replication analysis using an additional set of harmonized instruments. The harmonized instruments used in the univariable MR analyses for replication analysis are detailed in Supporting Table 3.

The characteristics of instrumental variables associated with NAFLD, including the SNP identifier, chromosome, position, alleles, effect allele frequency, and estimated effect size, are detailed in Supporting Table 4.

### 2.4. Power Calculation

To assess instrumental strength, we calculated the proportion of trait variance explained by genetic instruments (*R* ^2^) and instrument strength (*F* statistic) according to the following formulas:

(1) F=N−K−1·R2K·1−R2,

where *R* ^2^ represents the proportion of variance explained by the genetic variants, *N* represents the sample size, and *K* represents the number of included SNPs.

(2) R2=∑1K2β21−EAF·EAF,

where EAF is the effect allele frequency and *β* is the estimated effect on mental disorders. Strong instruments were considered to be those with an *F* statistic over 10, which could alleviate the bias caused by weak instruments [29]. We also calculated the statistical power of this study based on the mRnd website (https://shiny.cnsgenomics.com/mRnd/). The complete summary of IV information is provided in Supporting Table 5.

### 2.5. Statistical Analyses

LDSC regression analysis [30] was used to identify shared genetic architecture and estimate the genetic contributions between complex diseases and complex traits. Initially, we used LDSC regression analysis to explore the association between mental disorders and NAFLD preliminarily. Subsequently, we conducted the MR analysis. The main MR analysis utilized the multiplicative random effects inverse‐variance weighted (IVW) model, which assumes all SNPs are valid instruments and provides the most accurate estimation [31]. Estimates based on different data sources were combined using the fixed‐effects meta‐analysis method. To estimate heterogeneity across studies, *I* ^2^ statistics and their corresponding confidence intervals (CIs) were calculated. To evaluate the robustness of the IVW estimates under different assumptions, MR‐Egger, weighted median, weighted mode, and simple mode methods were used in sensitivity analyses. MR‐Egger is used to identify and adjust for potential directional pleiotropic bias, but its precision is limited [32]. Weighted median methods produce consistent causal estimates if more than half of the instrumental variables used are valid [33]. Weighted mode and simple mode methods cluster SNPs based on similar causal effects and estimate causal effects based on the largest cluster [34]. MR results that were unanimous across all five methods were considered reliable estimates of association [35]. In addition, causal analysis using summary effect estimates (CAUSE) was used to avoid false‐positive results caused by horizontal pleiotropy [36]. MR–Robust adjusted profile score (MR‐RAPS), which can provide a robust statistical estimate for our MR study even with several weak IVs, was used to enhance statistical power and address the issue of horizontal multicollinearity [37].

In the MR analysis, multiple testing was conducted, and a Bonferroni‐corrected threshold of *p* values under 6.25 × 10^−3^ (*p* = 0.05/8), that is, eight exposures and one outcome in forward MR analysis, as well as one exposure and eight outcomes for reverse MR analysis, were regarded as strong evidence of a causal association for pooled main IVW results, while *p* values under 0.05 but above 6.25 × 10^−3^ were considered suggestive of an association. Cochran’s *Q* value was used to measure heterogeneity among SNP estimates. The MR–pleiotropy residual sum and outlier (MR‐PRESSO) method can identify outliers by removing SNPs that contribute disproportionately more than expected to the heterogeneity. The MR‐PRESSO [38] and RadialMR [39] were used to identify outliers in IVW and MR–Egger regression models. Finally, genetic variants that are more strongly associated with the outcome than the exposure were identified and excluded using Steiger‐filtering analyses [40].

Mediation analysis is designed to assess the pathway from exposure to outcome through an intermediary variable, thereby elucidating the potential mechanisms through which exposure influences the outcome [41, 42]. For the robust and reliable causal relationships identified in the preliminary analysis, mediation analysis was performed to examine potential mediating factors from mental disorders to NAFLD [43]. Specifically, our mediation analysis focused on established causal factors of NAFLD, encompassing T2DM (glycated hemoglobin [HbA1c], fasting glucose, fasting insulin), obesity (body mass index [BMI], body fat percentage, waist circumference, waist‐to‐hip ratio [WHR]), hypertension (diastolic blood pressure, systolic blood pressure), and dyslipidemia (triglycerides [TG], total cholesterol, high‐density lipoprotein cholesterol [HDL‐C], low‐density lipoprotein cholesterol [LDL‐C], very‐low‐density lipoprotein cholesterol [VLDL‐C]; concentration of VLDL, LDL, and HDL particles in different sizes; and cholesterol to total lipids ratio in VLDL, LDL, and HDL particles in different sizes). The methodology involved an initial step where the causal relationships between mental disorders and the aforementioned 23 factors were evaluated using two‐sample MR methods to determine the beta coefficient (βA). Subsequently, multivariable Mendelian randomization (MVMR) was employed to identify which of the 23 factors retained a causal relationship with NAFLD after adjusting for mental disorders, yielding another beta coefficient (βB) and ensuring the mediating effects on outcomes were independent of exposure [41]. The mediation effect was quantified using a two‐step MR approach: mediation effect = βA × βB. The total effect of mental disorders on NAFLD, established in the previous two‐sample MR analysis, was isolated by calculating the direct effect as (total effect − mediation effect). The proportion of the effect mediated was determined by the formula: mediation proportion = (mediation effect/total effect) × 100%. The 95% CIs for the mediation effects and the proportion of the effect mediated were estimated using the delta method [41]. Additionally, colocalization analysis was used to assess the posterior probability of whether the same genetic variants are responsible for the two GWAS signals.

Basic analysis in MR was conducted using the “Two Sample MR,” “Mendelian Randomization,” “MRPRESSO,” “mr.raps,” and “Radial MR.” The “meta” package was used for meta‐analysis of IVW results. The “cause” package was used to conduct CAUSE analyses. The LD Scores were calculated using “ldsc,” and colocalization analyses were done using “coloc R.” All analyses were conducted in R software (Version 4.3.0).

## 3. Results

### 3.1. Causal Effects of Mental Disorders on NAFLD

**Figure 2:**
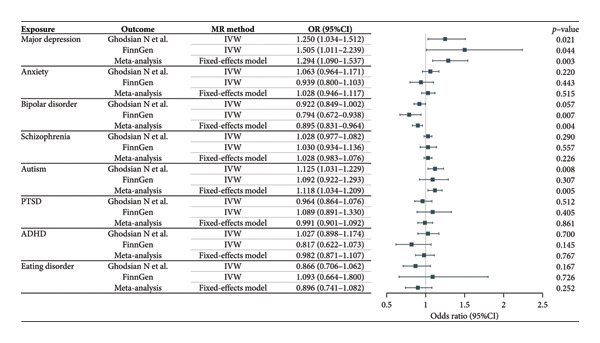
Associations of mental disorders with the occurrence of NAFLD in discovery, replication, and combined datasets. Estimates were obtained from the inverse‐variance weighted method. OR, odds ratio; CI, confidence interval; IVW, inverse‐variance weighted; PTSD, post‐traumatic stress disorder; ADHD, attention deficit hyperactivity disorder.

To explore the causal relationship between the eight mental disorders and NAFLD, we first conducted MR analysis to explore the potential causal effect of mental disorders on NAFLD (Figure 2 and Supporting Table 6). Among these, major depression, bipolar disorder, and autism showed a significantly positive association with NAFLD occurrence in pooled IVW‐meta results. These associations remained significant after multiple comparison corrections [odds ratio (OR) = 1.294, CI: 1.090–1.537, *p* = 0.003 for major depression]. Additionally, our initial exploratory analyses identified potential associations for bipolar disorder (OR = 0.895, CI: 0.831–0.964, *p* = 0.004) and autism (OR = 1.118, CI: 1.034–1.209, *p* = 0.005). However, as detailed in the subsequent sensitivity analyses, these associations did not demonstrate sufficient robustness. No evidence of heterogeneity was found [*I* ^2^ = 0%, *p* = 0.41 for major depression; *I* ^2^ = 60%, *p* = 0.12 for bipolar disorder; *I* ^2^ = 0%, *p* = 0.76 for autism]. In contrast, the other five mental disorders, including anxiety, PTSD, autism, and schizophrenia, did not exhibit significant causal associations with NAFLD occurrence in our primary analyses (Figure 2, Supporting Table 7). For the exploratory analyses of anxiety, PTSD, and autism, which utilized instruments selected under the relaxed significance threshold (*p* < 5 × 10^−6^), we assessed the instrument strength to address concerns of weak instrument bias. As detailed in Supporting Table 1, the mean F‐statistics for the instrument sets were 24.4 for anxiety, 23.1 for PTSD, and 24.3 for autism. As all F‐statistics substantially exceeded the conventional threshold of 10, the risk of bias from weak instruments was considered minimal.

### 3.2. Method Validation and Robustness Analysis

**Figure 3:**
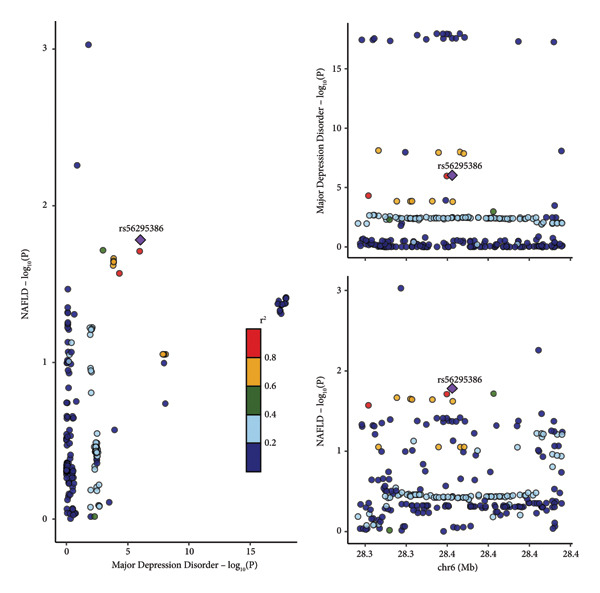
Regional LocusZoom plots and colocalization analyses results. Each point represents a variant with chromosomal position on the *x*‐axis (within 50‐kb regions of each sentinel variant for candidate proteins) and the −log10 (*p* value) on the *y*‐axis. Gene locations are shown at the bottom of the plot.

To further alleviate the influence of horizontal pleiotropy, we performed MR‐CAUSE analysis (Table 2). The results indicated that only the association between major depression and NAFLD was still statistically significant [OR = 1.203, CI: 1.082–1.338, *p* = 0.042] Sensitivity analyses using various methods, including MR‐Egger, weighted median, weighted mode, simple mode, and MR‐raps, confirmed the robustness of the observed associations (Supporting Table 7). Furthermore, to ensure the relaxed *p*‐value threshold for anxiety, PTSD, and autism did not bias our main conclusions, we conducted a sensitivity analysis excluding these traits; the putative causal association between major depression and NAFLD remained significant, underscoring the stability of this specific finding. We also conducted colocalization analysis between the GWAS for major depression and the NAFLD GWAS, revealing a low posterior probability of 10.5% in colocalization analysis. This suggests that the observed association is unlikely to be mediated by a single shared causal genetic variant at this locus, indicating that the potential causal pathway may be more complex or influenced by horizontal pleiotropy not fully captured by other sensitivity analyses (Figure 3).

**Table 2:** MR‐CAUSE analysis, linking genetically predicted mental disorders with NAFLD.

Exposure	Outcome	Model 1	Model 2	Delta_elpd	Se_delta_elpd	*z*	*p* value
Major depression	NAFLD	Null	Sharing	−7.16735111160124	3.42865609789477	−2.09042578402718	0.0182897843661818
		Null	Causal	−9.94550686827173	4.64221470123472	−2.14240562066775	0.01608042799758
		Sharing	Causal	−2.77815575667049	1.61027022082782	−1.72527301364505	0.0422391414523777

Attention deficit hyperactivity disorder (ADHD)	NAFLD	Null	Sharing	0.169417996518073	0.567109472231284	0.29873949354346	0.617430589604281
		Null	Causal	0.117051891912712	1.53408172571684	0.0763009492587599	0.530410164675895
		Sharing	Causal	−0.052366104605361	1.02038416075835	−0.0513199896854948	0.479535269813062

Anxiety disorder	NAFLD	Null	Sharing	0.00643541541801651	0.0806553575395051	0.0797890631736948	0.531797488543122
		Null	Causal	−0.413266688388955	1.23647386376029	−0.334230023376437	0.369102995012045
		Sharing	Causal	−0.419702103806972	1.15907713588553	−0.362100235448371	0.358638563853071

Post‐traumatic stress disorder (PTSD)	NAFLD	Null	Sharing	0.157165275036862	0.0513710450936801	3.05941362007053	0.99889114634537
		Null	Causal	0.899777307417045	0.206587174876269	4.35543642995239	0.999993359897264
		Sharing	Causal	0.742612032380183	0.169662797231981	4.3769880285824	0.999993983470963

Bipolar disorder	NAFLD	Null	Sharing	0.263728087802846	0.433511167703813	0.608353619122986	0.728523517033111
		Null	Causal	0.800717117118655	1.10008438191713	0.727868816502275	0.766653053165235
		Sharing	Causal	0.536989029315809	0.687069362466689	0.781564509568483	0.782764724432665

Autism spectrum disorder	NAFLD	Null	Sharing	−2.22659320196674	1.83842995081557	−1.21113845049085	0.112921173576526
		Null	Causal	−3.40996513338887	3.0348843782516	−1.12358980059509	0.130593542492832
		Sharing	Causal	−1.18337193142213	1.45546058621199	−0.813056665795396	0.208092785065342

Schizophrenia	NAFLD	Null	Sharing	0.315470176103556	0.437844118695851	0.720507967637445	0.764393852098303
		Null	Causal	0.726248834306532	1.14091612906819	0.636548836328291	0.737790619615765
		Sharing	Causal	0.410778658202976	0.71830351285085	0.571873380616852	0.716296120418137

Eating disorders	NAFLD	Null	Sharing	0.38550213157604	0.148811438766728	2.59054098778213	0.99520873952404
		Null	Causal	1.10697242822723	0.378442996432892	2.92507045621474	0.998278108785052
		Sharing	Causal	0.721470296651194	0.345236378430949	2.08978642381251	0.981681505281286

### 3.3. Reverse Effects of NAFLD on Mental Disorders

Additionally, we performed reverse MR analysis to explore the potential causal effect of NAFLD on mental disorders (Supporting Table 8). Our results showed no significant causal effect of genetically predicted NAFLD on any of the eight mental disorders (Supporting Table 9). To assess the reliability of these null findings, we calculated the F‐statistics for the instrumental variables and the statistical power of our analysis. The F‐statistic for the NAFLD instruments was sufficiently high (*F* > 10), indicating strong instruments and a low risk of weak instrument bias. However, the statistical power to detect a potential causal effect (assuming a true OR of 1.2) varied across the outcomes (Supporting Table 9). For instance, the power to detect an effect on ADHD (5.0%) and bipolar disorder (8.7%) was limited, meaning a weak causal relationship cannot be definitively ruled out for these conditions based on our analysis. Conversely, the analysis for autism spectrum disorder had high power (84.1%), providing more robust evidence for a true null effect. For other disorders like major depression, the power was modest (29.3%). Overall, while our study does not support NAFLD as a major driving factor for these mental disorders, the interpretation of the null results should be cautious, especially for outcomes with low statistical power.

### 3.4. Genetic Correlations and Mediation Mechanisms

To assess the genetic correlations between mental disorders and NAFLD, we performed LDSC regression analysis using comprehensive GWAS summary data (Table 3). The analysis revealed significant positive associations for certain disorders with NAFLD, including major depression [genetic correlation (Rg) = 0.538, *p* = 1.36*E* − 07], ADHD [Rg = 0.798, *p* = 1.10*E* − 08], and PTSD [Rg = 0.579, *p* = 0.009], indicating potential shared genetic etiology. The other five diseases were not significantly associated with NAFLD occurrence (Supporting Table 6).

**Table 3:** Genetic correlation estimates from LDSC regression in mental disorders.

Exposure	Outcome	Rg	SE	*p* value
Major depression	NAFLD	0.538	0.102	1.36E‐07
Attention deficit hyperactivity disorder (ADHD)	NAFLD	0.798	0.140	1.10E‐08
Anxiety disorder	NAFLD	0.350	0.345	0.310
Post‐traumatic stress disorder (PTSD)	NAFLD	0.579	0.220	0.009
Bipolar disorder	NAFLD	0.152	0.098	0.120
Autism spectrum disorder	NAFLD	0.271	0.140	0.010
Schizophrenia	NAFLD	0.168	0.092	0.069
Eating disorders	NAFLD	−0.139	0.128	0.278

*Note:* LDSC: linkage disequilibrium score; Rg: genetic correlation; SE: the standard error of Rg.

**Figure 4:**
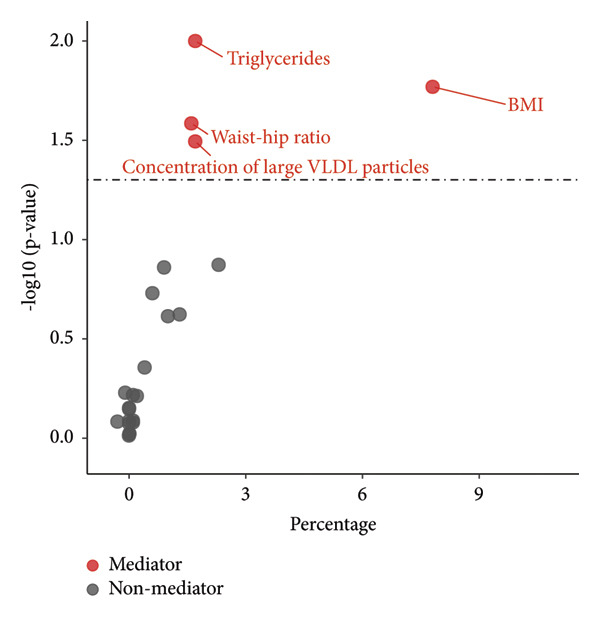
Evidence for selection of mediators in the association between mental disorders and NAFLD. The color red represents statistically significant mediating factors, while gray represents nonstatistically significant mediating factors. BMI, body mass index.

To explore the underlying mechanism behind the causal effect of major depression on NAFLD incidence, we further conducted a mediation analysis to assess potential mediators. The candidate potential mediators are chosen from known causal factors of NAFLD, including T2DM (HbA1c, fasting glucose, fasting insulin), obesity (BMI, body fat percentage, waist circumference, WHR), hypertension (diastolic blood pressure, systolic blood pressure), and dyslipidemia (TG, total cholesterol, HDL‐C, LDL‐C, VLDL‐C; concentration of VLDL, LDL, and HDL particles in different sizes; cholesterol to total lipids ratio in VLDL, LDL, and HDL particles in different sizes). Further mediation analysis identified BMI (39.33%; [95%CI: 37.50%‐81.09%], *p* = 0.017), WHR (8.09%; [95% CI: 7.99%‐10.21%], *p* = 0.026), TG (39.33%; [95%CI: 37.50%–81.09%], *p* = 0.017), and serum concentration of large VLDL particles (8.76%; [95%CI:7.53%–8.81%], *p* = 0.032) as mediators in the causal pathway from major depression to NAFLD incidence, suggesting these factors may underpin the observed associations (Figure 4 and Supporting Table 9).

## 4. Discussion

In this study, we performed a comprehensive two‐sample MR study to investigate the association of mental disorders with NAFLD. Among the eight mental disorders we studied, our primary analyses suggested potential links between major depression, bipolar disorder, and autism with NAFLD occurrence. However, after applying more stringent sensitivity and robustness analyses, only the putative causal effect of major depression on NAFLD was consistently supported. In reverse MR analysis, we found no causal effect of NAFLD on any mental disorders. In further investigation of the causal pathway from major depression to NAFLD, we identified the cholesterol‐to‐total‐lipids ratio in large LDL and the concentration of large VLDL particles as causal mediators, which mediated 9.24% and 8.76% of the risk from major depression to NAFLD occurrence, respectively.

Previous observational studies have indicated an association between major depression and NAFLD, with a notably higher prevalence of major depression—nearly 30%—in individuals with NAFLD compared to the general population [44]. NAFLD has been identified as an independent risk factor for the occurrence of major depression [16], and the presence of NAFLD is associated with an increase in depression severity [45]. Conversely, major depression is linked to more severe hepatocyte ballooning in patients [46]. These studies collectively indicated an association of the occurrence of major depression and NAFLD, but the presence and direction of a causal relationship between them are not clear. By using MR, our study identified a causal effect of major depression on NAFLD occurrence, supporting the observational findings. Interestingly, an observational study with follow‐up data showed that NAFLD patients with comorbid major depression had a poorer response to standard NAFLD care than those without depression [47], emphasizing the impact of depression on NAFLD treatment outcomes.

The mechanism for the causal effect of major depression on NAFLD occurrence has not been fully elucidated. The pathogenesis of NAFLD is complicated and involves various irregularities in metabolism, the immune system and the endocrine system [48, 49]. Major depression can trigger systemic dysregulations such as MetS and chronic low‐grade inflammation, which exacerbate both NAFLD and depression [50–52]. Therefore, abnormal cytokines and metabolites produced by neuro‐inflammation in major depression may transmit to the liver through the “brain–liver” axis and facilitate the onset and development of NAFLD [53, 54].

Chronic psychological stress, a common feature of psychiatric disorders, activates the body’s stress response systems, particularly the HPA axis, which is crucial for lipid metabolism [17, 18]. Research indicates that the HPA axis is necessary for liver fat synthesis and that exposure to stressors, such as low‐intensity noise, can exacerbate NAFLD by this pathway [55, 56]. Furthermore, chronic stress can elevate glucocorticoid levels, leading to increased inflammatory factors and gut permeability changes, creating a vicious cycle that contributes to NAFLD development [57]. Moreover, insulin resistance, closely linked to psychiatric disorders, is a key pathological feature of NAFLD. Studies have shown a negative correlation between insulin resistance and cognitive function [58] and a higher risk of elevated blood sugar levels in offspring of parents with psychiatric disorders [59]. This resistance can lead to abnormal blood sugar control and promote liver fat deposition, further implicating psychiatric disorders in NAFLD pathogenesis [60].

Notably, recent research has revealed that the inactivation of neurons in the dorsal motor nucleus of the vagus nerve (DMV) reduces intestinal fat absorption, while activation of the DMV increases fat absorption [61]. Neuronal activation‐induced gut inflammation may contribute to the development of NAFLD, aligning with our findings on the impact of major depression on NAFLD [62]. The role of neuropeptide Y (NPY) in sustaining thermogenic fat and preventing obesity, as discussed in Zhu et al., suggests a potential mechanism where altered NPY signaling could influence metabolic processes contributing to the NAFLD development [63]. Given that psychiatric disorders, including depression, have been associated with vagus nerve dysfunction [64, 65], it is plausible that depression may influence intestinal fat absorption through the vagus nerve, thereby affecting NAFLD’s progression.

Lastly, psychiatric disorders are associated with alterations in the gut microbiome, a critical factor in human health [20]. Dysbiosis of the gut microbiota in patients with psychiatric disorders can disrupt energy metabolism, increase endotoxin production, enhance intestinal permeability, and cause endotoxemia, all exacerbating liver inflammation and fat accumulation, contributing to NAFLD [66, 67]. While these mechanisms are promising, further research is needed to confirm how they interact between psychiatric disorders and NAFLD.

In our mediation analysis, two obesity‐related traits (i.e., BMI and WHR) were identified as mediators in the causal effect of major depression on NAFLD occurrence. BMI is universally regarded as a predictor of whole‐body fat percentage, whilst WHR is used for prediction of visceral adipose tissue mass in assessment of central obesity [68]. Obesity, especially central obesity, is one of the major risk factors for NAFLD incidence [69]. Observational studies have shown positive associations between BMI and major depression, particularly in females, and higher WHR has been linked to a greater prevalence of depressive symptoms [70–72]. Major depression, especially the atypical subtype, may contribute to the abnormal increase in visceral adipose tissue, as reflected by elevated WHR and BMI, through complex neural and hormonal mechanisms, thus facilitating NAFLD development [73–75]. This highlights the importance of considering female and atypical major depression patients in further research. Additionally, our analysis has revealed that TG and serum large VLDL particle concentration mediate the causal effect of major depression on NAFLD. VLDL, the primary lipoprotein for transporting TG in the serum, is implicated in NAFLD pathogenesis when its synthesis or secretion is disrupted, leading to liver TG accumulation. Hypertriglyceridemia is a known risk factor for NAFLD [76], and large VLDL levels are positively associated with NAFLD incidence [77–79]. In major depression, both serum TG and VLDL levels are increased, correlating with the severity of depression [80–83]. Recent findings suggest a potential causal effect of major depression on VLDL elevation in NAFLD patients [84, 85]. Large VLDL, in particular, is more strongly associated with metabolic dysregulations such as atherosclerosis, insulin resistance, and diabetes mellitus, indicating its unique role in these conditions [86]. VLDL may also influence NAFLD through its impact on insulin resistance or by affecting oxidative stress, as evidenced by research showing that VLDL uptake and lipid deposition are associated with increased oxidative stress and diminished insulin sensitivity [87].

The study’s strength lies in its robust methodology, utilizing large‐scale GWAS data and various statistical methods to ensure reliable results. Our study, while insightful, has limitations. The absence of certain GWAS data means potential mediators like psychomotor retardation and dietary habits between depression and NAFLD might not have been fully considered. The study’s focus on European populations limits the applicability of results to other ethnicities. Additionally, without stratified data for factors like gender and age and detailed subgroup analyses, the generalizability of our findings is uncertain. Another significant limitation is the impact of pharmacological treatments for mental disorders on liver function and NAFLD development, which was not accounted for. Furthermore, our findings are based on summary‐level GWAS data from individuals of European ancestry, limiting their generalizability to other ethnicities. While established sex differences exist in both major depression [88] and NAFLD [89], the lack of publicly available, stratified GWAS data precluded subgroup analyses by sex or age. Future studies using individual‐level data are needed to explore these potential heterogeneities.

A particularly significant limitation, as astutely noted, is the inability to account for the impact of pharmacological treatments. Many psychiatric medications, such as certain antidepressants and antipsychotics, are well documented to influence metabolic health by inducing weight gain, altering lipid profiles, and impairing insulin sensitivity [90], all of which are established risk factors for NAFLD [91]. Consequently, medication use acts as a major unmeasured confounder and a potential mediator in the causal pathway from mental disorders to NAFLD. The summary‐level GWAS data used in our analysis do not contain information on individual medication status, precluding our ability to perform stratified analyses or adjust for this crucial factor. Future research utilizing individual‐level data with detailed records on psychiatric medication is therefore essential to disentangle the direct effects of the mental disorders from the iatrogenic effects of their treatment on NAFLD risk.

## 5. Conclusion

In conclusion, our findings suggest a potential causal contribution of major depression to NAFLD occurrence, as well as the importance of mental health screening in NAFLD patients and NAFLD screening in major depression patients. By recognizing the potential causal link between NAFLD and major depression, healthcare professionals can provide more holistic care and improve the overall well‐being of patients with NAFLD and/or major depression. At the same time, the identified mediating factors, such as central obesity and serum large VLDL, in our study should be given due attention to facilitate more effective intervention for NAFLD. Additionally, in future basic research, molecular mechanisms regarding major depression‐associated NAFLD and the brain–liver axis should be further investigated to deepen understanding of the pathogenesis of both NAFLD and major depression.

## Ethics Statement

The authors have nothing to report.

## Consent

The authors have nothing to report.

## Disclosure

All authors read and approved the final manuscript.

## Conflicts of Interest

The authors declare no conflicts of interest.

## Author Contributions

Yinshi Su and Shuangzhe Lin carried out the studies, participated in collecting data, and drafted the manuscript. Yinshi Su, Yuanwen Chen, and Yongnian Ding performed the statistical analysis and participated in its design. Jingping Xiong, Ling She, and Yan Zhang participated in the acquisition, analysis, or interpretation of data and drafted the manuscript. Yinshi Su, Shuangzhe Lin, and Ling She contributed equally to this work as co‐first authors.

## Funding

This study was supported by the National Nature Science Foundation of China (no.: 82270620) and Science and Technology Commission of Shanghai Municipality (no.: 22140901500).

## Supporting Information

Supporting Table 1: Characteristics of instrumental variables and F‐statistics for anxiety, PTSD, and autism.

Supporting Table 2: List of harmonized instrumental variables for the causal effect of mental disorders on NAFLD.

Supporting Table 3: Harmonized instruments used in the univariable MR analyses for replication analysis.

Supporting Table 4: Characteristics of instrumental variables associated with NAFLD.

Supporting Table 5: List of instrumental variables used for major depression.

Supporting Table 6: Summary of Mendelian randomization analyses of the causal effects of mental disorders on NAFLD.

Supporting Table 7: Sensitivity analysis for the causal effect of mental disorders on NAFLD.

Supporting Table 8: Summary of Mendelian randomization analyses of the causal effects of NAFLD on mental disorders.

Supporting Table 9: Results of mediation analysis for the causal pathway from major depression to NAFLD.

## Supporting information


**Supporting Information** Additional supporting information can be found online in the Supporting Information section.

## Data Availability

All data generated or analyzed during this study are included in the published article.
